# The effect of a direct-fed microbial (10-G) on live animal performance, carcass characteristics, and *Salmonella* prevalence of fed beef heifers

**DOI:** 10.1093/tas/txae086

**Published:** 2024-05-29

**Authors:** Travis J Tilton, Kevin Martens, Loni W Lucherk, Alyssa B Word, Ben P Holland, Ty E Lawrence, Travis C Tennant

**Affiliations:** Beef Carcass Research Center, West Texas A&M University, Canyon, TX 79016, USA; Life Products Inc., Norfolk, NE 68701, USA; Beef Carcass Research Center, West Texas A&M University, Canyon, TX 79016, USA; Cactus Research, Amarillo, TX, USA; Cactus Research, Amarillo, TX, USA; Beef Carcass Research Center, West Texas A&M University, Canyon, TX 79016, USA; Beef Carcass Research Center, West Texas A&M University, Canyon, TX 79016, USA

**Keywords:** cattle, feedlot, pathogen

## Abstract

The objective of this study was to determine the efficacy of the direct-fed microbial 10-G upon cattle growth performance, liver and lung health, carcass quality, and yield outcomes, as well as prevalence and enumeration of *Salmonella* in feces and lymph nodes. Fed beef heifers (*N* = 1,400; initial shrunk body weight [**BW**] 343.3 ± 36.2 kg) were blocked by the day of arrival and randomly allocated to one of two treatments (0 [negative control, CON] or 2 g of a direct-fed microbial [10-G] that provided 1 billion CFUs per animal per day of *Lactobacillus acidophilus*, *Enterococcus faecium*, *Pediococcus pentosaceus*, *L. brevis*, and *L. plantarum*) with 10 pens per treatment. Recto-anal mucosal fecal samples (**RAMs**; *n* = 477) and subiliac lymph nodes (**SLNs**; *n* = 479) were collected longitudinally at harvest from 23 to 25 heifers per pen. Data were analyzed using mixed models; pen served as the experimental unit; block and harvest date were random effects. No differences were detected in dry matter intake (*P* = 0.78), final BW (*P* = 0.64), average daily gain (*P* = 0.51), gain to feed (*P* = 0.71), hot carcass weight (*P* = 0.54), dressed carcass yield (*P* = 0.52), 12th rib fat depth (*P* = 0.13), longissimus muscle area (*P* = 0.62), calculated empty body fat (*P* = 0.26), or marbling score (*P* = 0.82). Distributions of liver scores (*P* ≥ 0.34), quality grades (*P* ≥ 0.23), and yield grades (*P* ≥ 0.11) were also not different between treatments. A tendency was detected for more normal lungs (*P* = 0.08; 10-G = 65.96%, CON = 61.12%) and fewer inflated lungs at harvest for cattle fed 10-G (*P* = 0.10; 10-G = 0.29%, CON = 1.16%); other lung outcomes did not differ (*P* ≥ 0.54). *Salmonella* prevalence did not differ for RAM samples (*P* = 0.41; 10-G = 97.74%, CON = 96.82%) or SLN (*P* = 0.22; 10-G = 17.92%, CON = 13.66%). *Salmonella* concentration of RAM samples (*P* = 0.25; 10-G = 3.87 log CFU/g, CON = 3.32 log CFU/g) or SLN (*P* = 0.37; 10-G = 1.46 log CFU/g, CON = 1.14 log CFU/g) also did not differ between treatments at harvest. These results do not demonstrate any difference in live animal performance, carcass characteristics, or *Salmonella* carriage for heifers fed 10-G.

## Teaser

Direct-fed microbials have been reported as a preharvest intervention for *Salmonella* with subtle improvements to growth performance metrics in finished cattle. However, our results displayed no effect on cattle performance or *Salmonella* carriage for heifers fed 10-G.

## INTRODUCTION

Even with major improvements to our food safety system in recent years, *Salmonella* remains a major foodborne pathogen estimated to cause 1.35 million cases of foodborne illness, 26,500 hospitalizations, and 420 deaths annually (CDC, 2023). Approximately 43% of those illnesses have been attributed to meat and poultry (Interagency Food Safety Analytics Collaboration, 2022), and 6% are attributed specifically to beef. *Salmonella* has been recovered from multiple areas of the beef carcass (Rhoades et al., 2009) but the most difficult to control with current postharvest interventions is *Salmonella* within the lymphatic system (Arthur et al., 2008; Brichta-Harhay et al., 2012; Haneklaus et al., 2012; Koohmaraie et al., 2012; Gragg et al., 2013) because lymph nodes are commonly incased in fat. Difficulty finding and removing all lymph nodes, particularly when lymph nodes are surrounded by fat tissue, increases the opportunity for unintentional incorporation into beef trimmings destined for ground beef. The grinding process then allows for *Salmonella* from infected lymph nodes to contaminate large batches of meat (Bosilevac et al., 2009; Koohmaraie et al., 2012). With rising pressure to declare *Salmonella* as an adulterant (USDA, 2023), preharvest interventions have been tested to reduce *Salmonella* prevalence and concentration. Direct-fed microbials (**DFMs**) are one such preharvest intervention that have been reported to reduce the prevalence of *Salmonella* by up to 13 percentage points in feces and up to 24 percentage points in lymph nodes when included in the fed cattle diet (Stephens et al., 2007; Vipham et al., 2015; Mayer et al., 2022). DFMs also have been reported to provide subtle growth performance benefits (Krehbiel et al., 2003; Cull et al., 2015) making them potentially advantageous for feedyards. However, other trials have reported no growth performance advantage (Elam et al., 2003; Stephens et al., 2010; Neuhold et al., 2012; Luebbe et al., 2013; Kenney et al., 2015; Wilson et al., 2016; Mayer et al., 2022), indicating inconsistent results from the feeding of DFMs. Thus, the objective of this study was to evaluate the effect of dietary inclusion of *Lactobacillus acidophilus*, *Enterococcus faecium*, *P. pentosaceus*, *L. brevis*, and *L. plantarum* upon feedlot growth performance, carcass outcomes, and *Salmonella* carriage of finished heifers.

## MATERIALS AND METHODS

All live animal experimental procedures followed the guidelines described in the Guide for the Care and Use of Agricultural Animals in Agricultural Research and Teaching (FASS, Savoy, IL).

### Cattle Processing

Heifers were received at a commercial feedyard in the Texas Panhandle between April 05, 2021 and April 26, 2021 from Oklahoma, Texas, and Mississippi. Prior to initial processing, cattle were penned together by source and were provided ad libitum access to water and coastal Bermuda grass hay. During initial processing, individual heifers were excluded from the trial if body weight (**BW**) deviated more than 68 kg from the mean pay weight of each arrival group or were deemed unfit due to illness, lameness, or pregnancy. Heifers were initially implanted with Revalor-IH (Merck Animal Health, Summit, NJ) and reimplanted (69 to 70 days on feed) with Revalor-200 (Merck Animal Health). Heifers were vaccinated against viral respiratory pathogens by administration of Titanium 3 (Elanco Animal Health, Indianapolis, IN) and Nasalgen IP (Merck Animal Health), and against bacterial respiratory pathogens by administration of Presponse SQ (Boehringer Ingelheim Vetmedica, Duluth, GA). For anti-parasiticide control, heifers were given Dectomax Injectable (Zoetis, Parsippany, NJ) and Synanthic (Boehriner Ingelheim Vetmedica).

### Experimental Design

Heifers (*N* = 1,400; initial BW 343.4 ± 36.2 kg) were enrolled in a generalized randomized block design with two treatments and 10 pen replications per treatment. Cattle were blocked by the time of arrival. Each arrival block (*N* = 5) contained four pens with two replications of each dietary treatment. Pens (*N* = 20) each housed 70 heifers. Heifers were randomly allocated to one of two treatments; a negative control (CON) or 2 g/animal/d (10-G) of 10-G Armor (Life Products, Inc., Norfolk, NE) to provide 1 billion CFUs per animal per day of *L. acidophilus*, *E. faecium*, *P. pentosaceus*, *L. brevis*, and *L. plantarum.* Individual BW was collected at initial processing and reimplant. Pen BW was collected on day 0 and prior to harvest on a platform scale (Model 7531, Mettler-Toledo, Columbus, OH) prior to morning feeding.

### Feed Delivery

Feed (Table 1) was delivered three times daily using a truck-mounted delivery box. Micro ingredients, excluding 10-G, were weighed using a micromachine (Micro Technologies, Amarillo, TX) and added directly to the feed batch. The additive 10-G was dispensed independently after the base ration had loaded into the delivery truck and was mixed for 3 min. Delivery trucks fed CON pens first, then 10-G pens, then something else on the feedyard. The quantity of 10-G dispensed from the micromachine was monitored daily relative to the quantity of 10-G diet made to ensure the dosing was correct. Feed bunks were checked three times daily at 0600 h, 1800 h, and 2100 h; the 0600 h evaluation served as the primary check. The starter diet was entirely RAMP (Cargill Corn Milling, Bovina, TX) for the first 10 days with the inclusion of 10-G for treatment pens. Transition to the finishing diet occurred over 28 d using a two-ration blending approach where systematic replacement of 10% to 15% of the daily feed call of RAMP was replaced with finishing ration. Increases in the amount of finish ration were made every 2 to 4 d. The finishing diet was prepared onsite in 3,628 kg batches. The ration included steam-flaked corn, Sweet Bran Plus (Cargill Corn Milling), wet distiller’s grains plus solubles, and other ingredients common to the cattle feeding region (Table 1). Additional feed additives included monensin sodium (Rumensin, Elanco Animal Health), tylosin phosphate (Tylan, Elanco Animal Health), melengesterol acetate (HeifermaX 500, Elanco Animal Health), and ractopamine hydrochloride (Optaflexx, Elanco Animal Health) for the final 31 d.

**Table 1.** Ingredient formulation and analyzed composition of starter and finishing diets^1^^,^^2^^,^^3^

Item	Starter		Finisher	
	CON	10-G	CON	10-G
Ingredient, %				
RAMP^4^	99.99	99.20	—	—
10-G, g/heifer	—	2.0	—	2.0
Steam flaked corn	—	—	52.99	52.96
Wet distillers grains	—	—	19.85	19.85
Sweet Bran Plus^5^	—	—	18.35	18.37
Fat^6^	—	—	1.37	1.38
Ground corn stalks	—	—	7.41	7.39
Micro ingredients				
Vitamin A, IU/lb	—	—	1,200	1,200
Vitamin D, IU/ lb	—	—	120	120
Monensin sodium, g/ton	20.0	20.0	42.0	42.0
Tylosin phosphate, g/ton	10.0	10.0	7.5	7.5
Melengestrol acetate, mg/ton	—	—	39.8	39.8
Ractopamine HCl, mg/ton	—	—	27.2	27.2
Analyzed composition, %				
Dry matter, %	60.5	59.8	60.1	59.5
Crude protein, %	21.5	21.3	15.5	15.3
Non-protein nitrogen, %	1.0	1.0	0.40	0.24
Neutral detergent fiber, %	40.0	40.5	24.4	25.1
Crude fiber, %	2.9	3.3	5.32	5.04
Calcium, %	1.6	1.5	0.85	0.78
Phosphorus, %	0.8	0.8	0.53	0.53
Magnesium, %	0.4	0.4	0.25	0.25
Potassium, %	1.6	1.6	0.85	0.85
Sulfur, %	0.4	0.4	0.21	0.21

^1^Treatments included no DFM contained in the diet (CON) and a diet containing *L. acidophilus*, *E. faecium*, *P. pentosaceus*, *L. brevis*, and *L. plantarum* fed at 2 g/heifer daily providing 1 × 10^9^ CFU (10-G; Life Products, Inc., Norfolk, NE).

^2^All values on a DM basis.

^3^Analysis and calculation performed by Servi-Tech Laboratories, Amarillo, TX.

^4^Complete starter feed (Cargill Corn Milling., Bovina, TX).

^5^Wet corn gluten feed (Cargill Corn Milling, Bovina, TX).

^6^Fat source was yellow grease from April 16, 2021 through June 30, 2021 and palm stearin from July 01, 2021 through September 27, 2021.

### Animal Accountability

Twenty heifers died or were removed during the study, 10 each from the CON and 10-G treatments. Nine heifers died, primarily diagnosed as respiratory or digestive disorders. Eleven heifers were removed from the study for failure to perform at the same rate as penmates.

### Sample and Data Collection

After 152 to 163 d on feed, heifers were transported 93 km to the beef processing facility (USDA Establishment M245E). Carcass data were collected by trained personnel from the West Texas A&M University Beef Carcass Research Center (Canyon, TX). Ear tag lot and individual ID were recorded and assigned a unique identification based on animal harvest order. Livers were individually scored using a modified Elanco Liver Check System (Brown and Lawrence, 2010; Lawrence, 2022). Each liver was scored based on the presence and severity of abscess (edible = no abscesses, A− = 1 or 2 small abscesses, A = 2 to 4 small active abscesses, A+ = 1 or more large active abscesses, A+ adhesion = liver adhered to gastrointestinal tract or diaphragm, A+ open = open abscess). Individual lungs were evaluated to determine the presence and severity of lung lesions using a modified system from Tennant et al. (2014). Lungs were examined for interlobular adhesions, missing tissue caused by plural adhesions, and tissue consolidation. Lung scores were N = normal; 1 = presence of mycoplasma-like lesion affecting <25% of lung tissue; 2 = plural adhesions causing a portion of lung to be missing and/or tissue consolidation affecting <25% of lung tissue; 3 = plural adhesions causing a portion of lung to be missing and/or tissue consolidation affecting 25% to 50% of lung tissue; 4 = plural adhesions causing a portion of lung to be missing and/or a combination of these affecting 50% to 75% of lung tissue; and 5 = plural adhesions causing a portion of lung to be missing and/or a combination of these affecting >75% of lung tissue. Lungs that were contaminated, inflated, or skipped were noted. Hot carcass weights (**HCWs**) were recorded on the harvest floor. Carcass characteristics (marbling, quality grade, 12th rib subcutaneous fat depth, longissimus muscle area, and yield grade) were obtained from vision camera data (VBG 2000, E+V Technology, Oranienberg, Germany).

### Recto-Anal Mucosal Sample, and Subiliac Lymph Node Collection

Candidate animals (*n* = 24) were randomly pre-identified for *Salmonella* sampling within each pen. Recto-anal mucosal samples (**RAMs**) and paired subiliac lymph nodes (**SLNs**) were collected at harvest. RAMs (*n* = 477) were obtained by palpation using OBSLEEVE’s (Agri-Pro Enterprises, IA). Samplers inserted their hand 10 to 20 cm into the recto-anal canal junction of each designated heifer and placed the entire palpation sleeve containing the fecal sample into sealable plastic containers with harvest order number correlated to individual heifer ID. The left or right SLN (*n* = 479) was collected from each sample animal at harvest. Lymph nodes were excised, kept intact and encased in fat, and placed in a bag with a label corresponding to the animal. All samples were placed on wet ice and shipped overnight to Food Safety Net Services (**FSNS**, San Antonio, TX) for diagnostic analysis.

### *Salmonella* Analysis

Upon arrival at FSNS, SLNs were categorized as small (<10 g) or medium (>10 g) size. Forty milliliters of prewarmed (42 °C) BAX MP media (Hygiena, Camarillo, CA, USA) were added to small SLNs, whereas 80 mL were added to medium SLN as the primary enrichment step. Excess fat and fascia were trimmed from SLN prior to being submerged in boiling water for 3 to 5 s, weighed, placed in a stomacher bag (Seward Model 400 or equivalent, Bohemia, NY) and pulverized. If the total weight was greater than 10 g, 80 mL of BAX MP media were used, if the SLN weight was less than 10 g, 40 mL of media were used. To achieve a homogenized sample, BAX MP media was added to the stomacher bag and hand massaged. Once homogenized, 3 mL of the solution were removed and incubated for 6 h at 42 °C (AOAC# 081201). Following incubation, samples were examined for *Salmonella* using a real-time PCR method (BAX System, Hygiena, Camarillo, CA). If the sample was negative, incubation was initiated for another 12 to 18 h and reexamined. Cycle threshold values from the BAX System, corresponding to each pathogen concentration, were documented and used in statistical analyses to estimate pathogen load. The results were then analyzed and quantified via the Hygiena Quant Calculator to determine Log_10_CFU/g.

### Statistical Analysis

Data were analyzed using the GLIMMIX procedure in SAS (version 9.4, SAS Institute, Cary, NC). The LSMEANS option was used to calculate the least square means. Pen served as the experimental unit. Treatment served as a fixed effect, and the random effect was block. Live animal growth performance outcomes were calculated on a deads and removals excluded basis. Significance was determined at *P* ≤ 0.05; tendencies were determined at *P* ≤ 0.10.

## RESULTS AND DISCUSSION

### Live Performance

Initial BW (Table 2) did not differ (*P* = 0.89) between treatment groups (CON = 331.9 kg vs. 10-G = 331.6 kg) nor did final BW (*P* = 0.64; CON = 570.4 kg, 10-G = 572.1 kg). Previous trials have also observed no difference in final BW (Elam et al., 2003; Stephens et al., 2010; Neuhold et al., 2012; Luebbe et al., 2013; Cull et al., 2015; Wilson et al., 2016; Smock et al., 2020; Mayer et al., 2022; Ryan et al., 2023) when cattle were supplemented with bacterial (*Bacillus subtilis*, *E. faecium*, *L. acidophilus*, *L. brevis*, *L. plantarum*, *P. acidilacticii*, and *Propionibacterium freudenreichii*) or yeast (*Saccharomyces cerevisiae*) DFM during the finishing period. In contrast, Krehbiel et al. (2003) in a review reported final BW was 4 to 7 kg heavier for cattle supplemented with varying concentrations of *L. acidophilus* (10^4^ or 10^6^ or 10^8^) and *Pr. freudenreichii* (10^8^ or 10^9^) when compared to controls.

**Table 2.** Live growth performance of heifers fed 10-G^1^

Item	CON	10-G	SEM	*P*-value
*n* pens	10	10	—	—
Initial body weight, kg	331.9	331.6	3.34	0.89
Final body weight, kg	570.4	572.1	4.60	0.64
Average daily gain, kg	1.51	1.53	0.06	0.51
Dry matter intake, kg	9.56	9.58	0.09	0.78
Gain to feed	0.159	0.159	0.002	0.71
Morbidity, %	1.14	1.43	—	0.64
Mortalities and removals, %	1.43	1.43	—	1.00

^1^Treatments included no DFM contained in the diet (CON) and a diet containing *L. acidophilus*, *E. faecium*, *P. pentosaceus*, *L. brevis*, and *L. plantarum* fed at 2 g/heifer daily providing 1 × 10^9^ CFU (10-G; Life Products, Inc., Norfolk, NE).

No difference was observed between treatments for average daily gain (**ADG**; *P* = 0.51; CON = 1.51 kg/d; 10-G = 1.53 kg/d), dry matter intake (**DMI**; *P* = 0.78; CON = 9.56 kg/d, 10-G = 9.58 kg/d), or gain-to-feed ratio (**G:F**; *P* = 0.71; CON = 0.159, 10-G = 0.159). Previous research has demonstrated that supplementing cattle with bacterial or yeast DFM did not alter DMI (Elam et al., 2003; Stephens et al., 2010; Neuhold et al., 2012; Luebbe et al., 2013; Cull et al., 2015; Kenney et al., 2015; Wilson et al., 2016; Smock et al., 2020; Mayer et al., 2022; Ryan et al., 2023) even though it is perceived that feeding a DFM alters the gut microflora toward more efficient bacteria (Krehbiel et al., 2003). Some studies have reported that DFM supplementation with *L. acidophilus* and *Pr. freudenreichii* has improved ADG (+0.04 to 0.05 kg/d; Krehbiel et al., 2003) or G:F (+0.04 kg:1 kg; Cull et al. 2015); however, most have reported no difference (Elam et al., 2003; Stephens et al., 2010; Neuhold et al., 2012; Luebbe et al., 2013; Kenney et al., 2015; Wilson et al., 2016; Smock et al., 2020; Mayer et al., 2022; Ryan et al., 2023) in ADG or G:F following supplementation with bacterial (*B. subtilis*, *E. faecium*, *L. acidophilus*, *L. brevis*, *L. plantarum*, *P. acidilacticii*, and *Pr. freudenreichii*) or yeast (*Sac. cerevisiae*) DFM.

Morbidity (*P* = 0.64; CON = 1.14%; 10-G = 1.43%) or mortality and railer rate (*P* = 1.00; CON = 1.43%, 10-G = 1.43%) did not differ between treatments. Morbidity and mortality outcomes are not often reported in DFM trials, thus comparisons to the current trial are few. No difference was also reported by Mayer et al. (2022), which fed the same DFM as the current trial. Likewise, Ryan et al. (2023) fed *B. subtilis* and did not detect any differences in the clinical health of feedlot steers. In contrast, Smock et al. (2020) reported a lower frequency of cattle treated once for bovine respiratory disease and reduced antimicrobial treatment costs for cattle-fed *B. subtilis*.

### Liver and Lung Outcomes

Liver scores (Table 3) were not altered (*P* ≥ 0.34) for heifers fed 10-G, which is similar to previous studies in which 10-G was supplemented (Neuhold et al., 2012; Luebbe et al., 2013) and similar to other studies that fed *L. acidophilus* and *Pr. freudenreichii* (Wilson et al., 2016) or *B. subtilis* (Smock et al., 2020; Ryan et al., 2023). Heifers had a relatively low liver abscess rate (CON = 7.03% and 10-G = 6.75%) compared to Brown and Lawrence (2010) database analysis of fed cattle carcass liver abnormalities (12%) or Herrick et al. (2022) which reported 20% abscess incidence in fed cattle. Total A+ abscesses were not different (*P* = 0.49) and low (CON = 2.00% and 10-G = 2.57%) relative to previous data on fed cattle which reported 9.6% total A+ abscesses (Herrick et al., 2022). Work by Mayer et al. (2022) did report a difference in total A+ abscess, with fewer abscesses in heifers fed 10-G. The percentage of edible livers, although not different between treatments (*P* = 0.62), was greater than the 69.2% rate reported by Herrick et al. (2022) but similar to the 82% rate reported by Brown and Lawrence (2010).

**Table 3.** Liver and lung outcomes at harvest of heifers fed 10-G

Item	TRT^1^		SEM	*P*-value
	CON	10-G		
Liver score^2^, %				
Edible	83.40	84.40	—	0.62
Abscessed	7.03	6.75	—	0.84
A−	4.20	3.19	—	0.34
A	0.87	1.02	—	0.78
A+	2.00	2.57	—	0.49
Flukes	0.60	0.60	—	0.99
Telangiectasis	0.14	0.28	—	0.58
Contamination	7.83	7.11	—	0.62
Lung score^3^, %				
Normal	61.12	65.96	—	0.08
One	10.09	9.11	—	0.54
Two	3.47	3.48	—	0.99
Three	2.82	2.40	—	0.63
Four	3.33	3.63	—	0.77
Inflated	1.16	0.29	—	0.10
Condemned	8.95	8.98	—	0.99

^1^Treatments included no DFM contained in the diet (CON) and a diet containing *L. acidophilus*, *E. faecium*, *P. pentosaceus*, *L. brevis*, and *L. plantarum* fed at 2 g/heifer daily providing 1 × 10^9^ CFU (10-G; Life Products, Inc., Norfolk, NE).

^2^Modified Elanco Liver Check System (Brown and Lawrence, 2010). Edible = no abscesses, A− = 1 or 2 small abscesses, A = 2 to 4 small active abscesses, A+ = 1 or more large active abscesses, A+ adhesion = liver adhered to the gastrointestinal tract, and A+ open = open liver abscesses.

^3^Lung scoring (Mayer et al., 2022): One = presence of mycoplasma-like lesion affecting <25% of lung tissue; Two = plural adhesions causing a portion of lung to be missing and/or tissue consolidation affecting <25% of lung tissue; Three = plural adhesions causing a portion of lung to be missing and/or tissue consolidation affecting 25% to 50% of lung tissue; Four = plural adhesions causing a portion of lung to be missing and/or a combination of these affecting 50% to 75% of lung tissue; Inflated = lungs that did not deflate.

Lungs were examined (Table 3) as a possible association between respiratory health and treatment. We detected a tendency for more normal lungs at harvest (*P* = 0.08; CON = 61.12%, 10-G = 65.96%) and fewer inflated lungs (*P* = 0.10; CON = 1.16%, 10-G = 0.29%) for heifers fed 10-G. Mayer et al. (2022) also reported inflated lungs occurred 1 percentage point less when cattle were supplemented 10-G, which may suggest a subtle improvement in lung health concomitant with 10-G supplementation. No difference in lung outcomes was detected when steers were fed *B. subtilis* (Smock et al., 2020). The percentage of healthy lungs was notably greater than previous outcomes by Tennant et al. (2014) which reported no more than 35.9% of lungs without lesions across all treatments. No other lung outcomes were affected by 10-G (*P* > 0.54) supplementation.

### Carcass Outcomes

Heifers fed 10-G were not different in HCW (Table 4; *P* = 0.54; CON = 368.7 kg, 10-G = 370.1 kg). Numerous studies have reported no difference in HCW for cattle supplemented with a bacterial (*B. subtilis*, *E. faecium*, *L. acidophilus*, *L. brevis*, *L. plantarum*, *P. acidilacticii*, and *Pr. freudenreichii*) or yeast (*Sac. cerevisiae*) DFM when compared to a control population (Elam et al., 2003; Stephens et al., 2010; Neuhold et al., 2012; Luebbe et al., 2013; Kenney et al., 2015; Wilson et al., 2016; Smock et al., 2020; Mayer et al., 2022; Ryan et al., 2023). However, contradictory outcomes have also been reported for DFM supplementation; Cull et al. (2015) reported that *L. acidophilus* and *Pr. freudenreichii* supplementation led to 0.37% less HCW whereas Krehbiel et al. (2003) reported 1.03% more HCW with varying concentrations of *L. acidophilus* and *Pr. freudenreichii*. Dressed yields were similar (*P* = 0.52; CON = 64.64%, 10-G = 64.69%) between treatments, which agrees with previous reports (Elam et al., 2003; Krehbiel et al., 2003; Stephens et al., 2010; Neuhold et al., 2012; Kenney et al., 2015; Wilson et al., 2016; Smock et al., 2020; Mayer et al., 2022; Ryan et al., 2023) for cattle supplemented with a bacterial (*B. subtilis*, *E. faecium*, *L. acidophilus*, *L. brevis*, *L. plantarum*, *P. acidilacticii*, and *Pr. freudenreichii*) or yeast (*Sac. cerevisiae*) DFM when compared to a control population. Calculated empty body fat (*P* = 0.26) marbling score (*P* = 0.82), longissimus muscle area (*P* = 0.62), and 12th rib fat thickness (*P* = 0.13) also were not different between treatments, similar to Neuhold et al. (2012), Luebbe et al. (2013), Kenney et al. (2015), Wilson et al. (2016), Smock et al., (2020), Mayer et al. (2022), and Ryan et al. (2023) which compared bacterial (*B. subtilis*, *E. faecium*, *L. acidophilus*, *L. brevis*, *L. plantarum*, *P. acidilacticii*, and *Pr. freudenreichii*) DFM to control populations. No difference in distributions of USDA quality grades (*P* ≥ 0.23) or yield grades (*P* ≥ 0.11) was observed between treatments, similar to outcomes reported by Stephens et al. (2010), Cull et al. (2015), Smock et al. (2020), and Ryan et al. (2023) who compared cattle supplemented with bacterial (*B. subtilis*, *E. faecium*, *L. acidophilus*, *L. brevis*, *L. plantarum*, *P. acidilacticii*, and *Pr. freudenreichii*) or yeast (*Sac. cerevisiae*) DFM to a control population. However, Mayer et al. (2022) did report a tendency for a greater percentage of Choice carcasses and fewer Select carcasses for cattle fed 10-G in the same region as the current study a year prior.

**Table 4.** Carcass performance of heifers supplemented with 10-G

Item	TRT^1^		SEM	*P*-value
	CON	10-G		
HCW, kg	368.7	370.1	2.64	0.54
Dressed yield, %	64.64	64.69	0.10	0.52
Longissimus muscle area, cm^2^	93.1	93.3	1.03	0.62
Marbling score^2^	467	468	6.0	0.82
12th rib fat thickness, cm	1.70	1.77	0.05	0.13
Empty body fat^3^, %	30.82	31.13	0.26	0.26
Quality grade, %				
Prime	2.48	3.67	—	0.23
Choice	70.24	67.69	—	0.32
Select	24.45	26.99	—	0.30
Lower grade^4^	1.34	0.84	—	0.23
Yield grade, %				
YG 1	12.34	10.22	—	0.23
YG 2	34.30	34.83	—	0.84
YG 3	39.65	38.75	—	0.74
YG 4	11.04	14.10	—	0.11
YG 5	2.29	1.72	—	0.47

^1^Treatments included no DFM contained in the diet (CON) and a diet containing *L. acidophilus*, *E. faecium*, *P. pentosaceus*, *L. brevis*, and *L. plantarum* fed at 2 g/heifer daily providing 1 × 10^9^ CFU (10-G; Life Products, Inc., Norfolk, NE).

^2^300 = Slight^00^, 400 = Small^00^, 500 = Modest^00^, 600 = Moderate^00^.

^3^Calculated empty body fat, % = 17.76107 + (4.68142 × 12th rib fat thickness, cm) + (0.01945 × HCW, kg)−(0.06754 × longissimus muscle area, cm^2^) + (0.81855 × QG); where 4 = Select, 5 = Choice-, 6 = Choice, 7 = Choice+, 8 = Prime (Guiroy et al., 2001).

^4^Lower grade includes No Roll, Commercial, and Dark cutting carcasses.

### Fecal Samples

*Salmonella* prevalence of RAM samples (Table 5) did not differ (*P* = 0.41) between cattle-fed CON (96.82%) and 10-G (97.74%) supplemented diets. These results are similar to those of Mayer et al. (2022), but especially high relative to other previous literature; Fedorka-Cray et al. (1998) reported that 38% of sampled fecal pats from 100 feedyards were positive for *Salmonella*. These data suggest cattle in the current study were exposed to extremely high levels of *Salmonella*. Studies evaluating DFM for control of *Salmonella* have been characterized by inconsistent results in reducing prevalence rates. Brown et al. (2020) also reported no difference in qualitative culture of fecal pats from pen surface for cattle fed either a *L. acidophilus* and *P. acidilactici* or *L. reuteri* with other *Lactobacillis* strains relative to control fecal pats. Similarly, Tabe et al. (2008) reported that fecal grabs of cattle fed *L. acidophilus* and *Pr. freudenreichii* had *Salmonella* levels equal to control animals. In contrast, Stephens et al. (2007) reported less fecal *Salmonella* for cattle receiving high doses of *L. acidophilus* and *Pr. freudenreichii*. Mean Log_10_CFU/g of RAMs positive for *Salmonella* (*P* = 0.25) was not different between CON and cattle fed 10-G (CON = 3.32 Log_10_CFU/g; 10-G = 3.87 Log_10_CFU/g).

**Table 5.** *Salmonella* prevalence and concentration of fecal samples and SLNs collected at harvest from heifers supplemented with 10-G

Item	TRT^1^		SEM	*P*-value
	CON	10-G		
Fecal *Salmonella* prevalence, %	96.82	97.74	—	0.41
Fecal *Salmonella* log, CFU/g (mean of positive samples)	3.32	3.87	0.34	0.25
SLN *Salmonella* prevalence, %	13.66	17.92	—	0.22
SLN *Salmonella* log, CFU/g (mean of positive samples)	1.14	1.46	0.35	0.37

^1^Treatments included no DFM contained in the diet (CON) and a diet containing *L. acidophilus*, *E. faecium*, *P. pentosaceus*, *L. brevis*, and *L. plantarum* fed at 2 g/heifer daily providing 1 × 10^9^ CFU (10-G; Life Products, Inc., Norfolk, NE).

### Lymph Nodes

*Salmonella* prevalence from SLNs was also not different (*P* = 0.22) for cattle fed 10-G (CON = 13.66% vs. 10-G = 17.92%). In contrast, Vipham et al. (2015) did report an 18.9 to 24.0 percentage point reduction in *Salmonella* prevalence in the SLN of cattle-fed *L. acidophilus* and *Pr. freudenreichii*. Mayer et al. (2022) also reported an 8.4 percentage point lower *Salmonella* prevalence in the SLNs of cattle fed 10-G in the same feeding region. Haneklaus et al. (2012) reported extreme variation for *Salmonella* lymph node prevalence of 0% to 100% depending on feedyard. Overall lymph node *Salmonella* prevalence in our study was much greater than that of Arthur et al. (2008) who only reported 1.05% *Salmonella*-positive lymph nodes in fed cattle. No difference was observed in Log_10_CFU/g for *Salmonella*-positive samples (*P* = 0.37; CON—1.14, 10-G—1.46), which is in agreement with Mayer et al. (2022). In contrast, Vipham et al. (2015) reported approximately 0.8 Log_10_CFU/g lower concentration of *Salmonella* in lymph nodes of cattle supplemented with *L. acidophilus* and *Pr. freudenreichii*.

## CONCLUSION

The cattle feeding industry has accepted the use of DFMs because they have sometimes demonstrated subtle improvements to growth performance metrics. However, feeding 10-G in the current study did not improve feedlot performance. Additionally, previous work has demonstrated an increase in quality grade while simultaneously reducing liver abscess rates. However, there were no differences between DFM treatment and control pens for any carcass parameter. Lastly, the combination of *L. acidophilus*, *E. faecium*, *P. pentosaceus*, *L. brevis*, and *L. plantarum* did not alter *Salmonella* prevalence or concentration of fecal or SLNs samples. In conclusion, these results do not demonstrate any improvement in live animal performance, carcass characteristics, or reduction in *Salmonella*, for heifers fed 10-G.
